# Melatonin, a Full Service Anti-Cancer Agent: Inhibition of Initiation, Progression and Metastasis

**DOI:** 10.3390/ijms18040843

**Published:** 2017-04-17

**Authors:** Russel J. Reiter, Sergio A. Rosales-Corral, Dun-Xian Tan, Dario Acuna-Castroviejo, Lilan Qin, Shun-Fa Yang, Kexin Xu

**Affiliations:** 1 Department of Cell Systems and Anatomy, UT Health, San Antonio, TX 78229, USA; tan@uthscsa.edu (D.-X.T.); qinlilan1980@gmail.com (L.Q.); 2 Centro de Investigacion Biomedica de Occidente, Del Instituto Mexicano del Seguro Social, Guadalajara 44340, Mexico; espiral17@gmail.com; 3 Centro de Investigacion Biomedica, Universidad de Granada, Granada 18916, Spain; dacuna@ugr.es; 4 Institute of Medicine, Chung Shan, Medical University, Taichung 40201, Taiwan; ysf@csmu.edu.tw; 5 Department of Molecular Medicine, UT Health, San Antonio, TX 78229, USA; xuk3@uthscsa.edu

**Keywords:** ionizing radiation, antioxidant, free radicals, apoptosis, angiogenesis, molecular mechanisms, invasion, breast, prostate, melatonin receptors, chemotherapy

## Abstract

There is highly credible evidence that melatonin mitigates cancer at the initiation, progression and metastasis phases. In many cases, the molecular mechanisms underpinning these inhibitory actions have been proposed. What is rather perplexing, however, is the large number of processes by which melatonin reportedly restrains cancer development and growth. These diverse actions suggest that what is being observed are merely epiphenomena of an underlying more fundamental action of melatonin that remains to be disclosed. Some of the arresting actions of melatonin on cancer are clearly membrane receptor-mediated while others are membrane receptor-independent and involve direct intracellular actions of this ubiquitously-distributed molecule. While the emphasis of melatonin/cancer research has been on the role of the indoleamine in restraining breast cancer, this is changing quickly with many cancer types having been shown to be susceptible to inhibition by melatonin. There are several facets of this research which could have immediate applications at the clinical level. Many studies have shown that melatonin’s co-administration improves the sensitivity of cancers to inhibition by conventional drugs. Even more important are the findings that melatonin renders cancers previously totally resistant to treatment sensitive to these same therapies. Melatonin also inhibits molecular processes associated with metastasis by limiting the entrance of cancer cells into the vascular system and preventing them from establishing secondary growths at distant sites. This is of particular importance since cancer metastasis often significantly contributes to death of the patient. Another area that deserves additional consideration is related to the capacity of melatonin in reducing the toxic consequences of anti-cancer drugs while increasing their efficacy. Although this information has been available for more than a decade, it has not been adequately exploited at the clinical level. Even if the only beneficial actions of melatonin in cancer patients are its ability to attenuate acute and long-term drug toxicity, melatonin should be used to improve the physical wellbeing of the patients. The experimental findings, however, suggest that the advantages of using melatonin as a co-treatment with conventional cancer therapies would far exceed improvements in the wellbeing of the patients.

## 1. Introduction

In 2004, a brief commentary was published related to the then-explored mechanisms by which melatonin modulates cancer initiation and progression [1]. In that summary, several processes by which melatonin may protect against cancer risk were acknowledged. The first of these related to cancer initiation where melatonin, because of its radical scavenging actions [2,3,4,5,6], prevents damage to nuclear DNA [7,8,9,10] that results when it encounters reactive oxygen or nitrogen species [11,12,13]. DNA mutilated by any means can undergo mutation and proceed to cancer [14,15]; such damage persistently occurs, and if it goes unrepaired, it continues to accumulate throughout a life time and is likely a primary cause of cancer in the elderly [16].

At the time that brief article was written, melatonin had been also reported to employ several means to restrain cancer promotion. For example, Blask et al. [17] had found that, via processes involving one of the two known classic membrane receptors (MT1) [18,19], melatonin limited the cellular uptake of a growth-promoting fatty acid, i.e., linoleic acid (LA). After its entrance into the cell, LA is converted to 13-hydroxyoctadecadienoic acid (13-HODE) which causes a series of intracellular events that culminate in cancer cell proliferation. Some of these events involve epithelial growth factor (EGF), the phosphorylation and stimulation of downstream signaling molecules, and the mitogen-activated kinases, mitogen-activated protein kinase kinase (MEK) and Extracellular signal-regulated kinase (ERK) 1 and 2. These findings attracted interest since LA, an *n*-6 fat, is in much higher concentrations in modern-day diets compared to that of our ancestors and the incidence of certain cancer types is increasing in areas of the world where *n*-6 fats are disproportionally-elevated in the diet [20,21]. Blask et al. [22] also mentioned that dietary melatonin, which had been identified in edible foods roughly a decade earlier [23,24], may be a factor in the reduction of cancer risk.

In addition to its ability to modify tumor growth by reducing LA uptake and metabolism, by 2004, it had also been found that melatonin inhibits telomerase activity and restrains the growth of MCF-7 human breast cancer xenografts growing in immunocompromised mice [25]. This enzyme is known to be essential for the synthesis of specialized ribonuclear proteins (the telomeres) that extend the ends of linear eukaryotic chromosomes. These molecular extensions are key for stabilizing chromosomal structure [26,27] and, as they shorten with each cell division as it occurs in normal cells [28,29], chromosomal structure is weakened leading to genetic instability and cellular aging. On the contrary, the usual upregulation of telomerase activity in cancer cells allows them to maintain DNA stability and contributes to their immortalization even though they undergo frequent mitoses. Thus, the observations that melatonin repressed telomerase activity in breast cancer cells has implications for the inhibition of this common cancer type.

The antiestrogenic action of melatonin could also enhance the ability of the indole to limit the proliferation of hormone-sensitive breast cancer. This action had been documented in experiments that used human breast cancer cells that possess the estrogen receptor α (ERα) and involved the MT1 membrane receptor and possibly also a nuclear binding site (RZRα) [30,31]. This effect of melatonin as an oncostatic agent generally appeared less robust than that resulting from a reduction in LA uptake, but across studies it was highly reproducible.

Finally, more indirect evidence identified yet another mechanism that could contribute to melatonin’s capacity to inhibit cancer growth. Kilic and coworkers [32], in a publication not involving cancer, had shown that melatonin markedly inhibits the levels of endothelin-1 (ET-1) in the brain of patients suffering from a stroke. ET-1, an important agent in promoting angiogenesis, had been implicated in the control of cancer enlargement [33]. ET-1 is often elevated in the plasma of patients with cancer [34]; it not only promotes the ingrowth of blood vessels, a process critical for cancer cells to obtain the required nutrients for rapid growth and for metastasis, ET-1 also protects cancer cells from undergoing apoptosis.

Based on the findings summarized above, at the conclusion of the 2004 commentary [1], use of melatonin was urged in clinical trials in cancer patients. At that time, there had been some uncontrolled, partially successful trials by Lissoni’s group [35,36,37] which suggested that melatonin would be useful in reducing cancer growth and in improving patient wellbeing but the data were highly fragmentary and inconclusive. In the interval between 2004 and the present, this field of research has expanded rapidly and the data today are essentially overwhelmingly convincing that melatonin has significant utility as an agent that negatively impacts multiple features of experimental cancers [38,39,40]. These data are reviewed herein and, again, the rationale for performing clinical trials with melatonin as a cancer therapy is patently obvious. This review does not evaluate all of the published reports that have confirmed melatonin’s capacity as an anticancer agent. Rather, papers were selected to illustrate the variety of tumor types modulated by melatonin and especially some of the proposed mechanisms involved.

## 2. Cancer Initiation: Genomic Damage and Instability

Genomic injury and rendering the nucleotide unstable contributes to the likelihood of a cell becoming cancerous. Given that these disturbances are a prelude to cancer, agents/procedures that weaken DNA structure participate in cancer initiation [41]. A large variety of agents unfortunately attack the genome and perturb its stability, increasing the probability of a mutation and cancer transformation.

### 2.1. Ionizing Radiation-Induced DNA Damage

**Figure 1:**
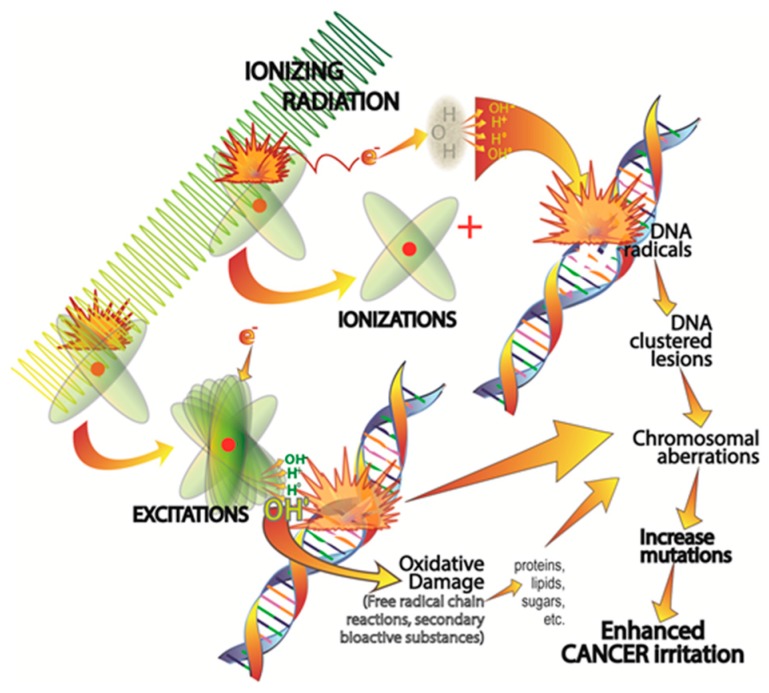
Chemical processes involved in ionizing radiation-mediated cellular injury as they relate to an elevated cancer risk. Clustered DNA damage and hydroxyl radical (•OH) generation are direct consequences of ionizing radiation exposure. The resulting damage to DNA in particular, but not exclusively, enhances the possibility of genetic mutations thereby boosting cancer initiation. Mitigating the damage to DNA and other molecules during Earth- and space-related high energy radiation exposure has important implications for health. As summarized in the text, melatonin given supplementally has significant ability to reduce molecular damage due to radiation exposure.

A major contributor to cancer development is ionizing radiation [42]. With regard to oncogenesis, the biological consequences of ionizing radiation are initially experienced at the genomic level when DNA absorbs energy. Such ionizing radiation occurs normally in nature and has been developed for therapies and other reasons. During ionizing radiation exposure, high energy photons or electrons are absorbed by DNA causing excitations or ionization with the latter being more likely to lead to mutations and tumor initiation (Figure 1) [43]. The ionization of molecules generates molecular species referred to as free radicals or reactive oxygen species (ROS) or nitrogen species (RNS) which often contain unpaired electrons making them highly reactive and damaging [44]. Free radicals/reactive species produced in the immediate vicinity of DNA induce its ionization which changes its biological functions. The damage resulting from exposure to ionizing radiation is referred to as its direct effect; if the damage goes unrepaired, the DNA may mutate [43]. In addition to the direct effects, the radiolytic products resulting from ionizing radiation exposure can cause molecular damage and serious physiological disruption. These are referred to as the indirect effects of ionizing radiation.

Agents which limit or quell ionizing radiation damage are identified as radioprotectors [45]. These exist naturally under endogenous circumstances and many have been synthetically fabricated. Some radioprotectors that cells produce include antioxidant enzymes, and low molecular weight free radical detoxifying agents and cytoprotectors, i.e., free radical scavengers. There are also a host of pharmaceutically-produced DNA protectors/scavengers, e.g., benzimidazoles, aminothiols, aminoglycosides [46], etc. Both the endogenously produced and industry-generated radioprotectors act in a variety of ways to limit DNA damage and cancer initiation including the direct detoxification of free radicals/reactive species, improving resistance against mutagenesis, stabilizing DNA, and promoting DNA repair. As DNA damage accumulates it can precipitate mutations which are conducive to malignant transformation.

### 2.2. Melatonin as a RadioProtector

Shortly after melatonin was discovered as a direct free radical scavenger [2,3], it was soon surmised that it would be useful as a radioprotector and anti-cancer agent [47]. This assumption was strengthened by the observations that melatonin highly effectively neutralizes the devastatingly toxic hydroxyl radical (•OH), which theoretically accounts for up to 70% of the genomic damage that occurs in cells exposed to high energy radiation. The pummeling of DNA by ionizing radiation produces a variety of damage, e.g., DNA single- and double-strand breaks, DNA-protein cross links, etc., all of which can result in a negative outcome in terms of cancer initiation [48].

That melatonin provides protection from ionizing radiation was initially documented in vivo by Blickenstaff and colleagues [47], the year following the discovery of melatonin as a •OH scavenger [2,49]. This group reported that half of mice irradiated with a normally lethal dose of ionizing radiation (950 cGy whole body) were protected from death when they were given melatonin (death was determined at 30 days after radiation exposure). Similarly, we [50] observed that melatonin also significantly improved 30-day survival of mice exposed to 815 cGy ionizing radiation. In an associated study, we found that melatonin given prior to whole body radiation exposure protected the highly sensitive bone marrow cells from genomic damage [51]. This observation has particular importance to the current discussion given that bone marrow cells are readily damaged by such radiation and the consequences in terms of blood cell dyscrasias is significant [52]. Vijayalaxmi and coworkers [53,54,55] published additional data verifying that melatonin serves as a cytoprotective agent of blood lymphocytes exposed to 150 cGy γ radiation. Based on the ability of melatonin to reduce the percentage of cells expressing genetic damage including acentric fragments, exchange type aberrations and micronuclei, it was justifiably deduced that melatonin functions as an effective radioprotector and resists DNA mutations and cancer initiation. These observations were extended to the human in an in vivo/ex vivo study [55]. In this case, human volunteers were given melatonin orally and thereafter circulating lymphocytes were collected and exposed to ionizing radiation ex vivo. Using this melatonin treatment paradigm, the frequency of chromosomal damage and micronuclei formation was reduced by 60–65% in the lymphocytes retrieved from individuals who were fed melatonin compared to similar cells collected from the daytime blood of non-melatonin-treated individuals. Also, importantly, melatonin, over a very wide range of doses, is devoid of cytotoxicity (in normal cells) and does not negatively impact mutation frequency [56,57,58].

The results of these and related studies documenting the protective actions of melatonin from ionizing radiation damage at the level of the genome have been extensively summarized elsewhere [59,60,61,62,63]. The ability of melatonin to protect nuclear DNA from free radical-mediated damage is consistent with the ability of melatonin to locate in the nucleus [64,65,66] and presumably in very close proximity to DNA where it would have to be to shield the nucleotide from the highly destructive •OH; this agent, because of its extremely high reactivity, always damages molecules in the immediate vicinity of where it is produced, i.e., the damage is “on site” [67,68].

The ability of melatonin to reduce DNA damage (and that to other molecules) surely derives, at least in part, from the direct scavenging actions of the parent molecule as well as that of its metabolites [69,70,71,72,73,74]. Additionally, this indole stimulates antioxidant enzymes that remove ROS before they can inflict damage [75,76,77,78,79] and aids in the repair of damaged DNA [80,81]. Each of these actions would limit the likelihood of DNA destruction and as a result, mutagenesis, thereby curtailing the likelihood of cancer.

The potential importance of melatonin in reducing cancer incidence due to its basic action as a radioprotector also has implications for its possible use under circumstances of prolonged low dose radiation exposure [82,83] such as occurred in Europe following the Chernobyl accident or in the event of the detonation of a “radiological dirty bomb” [84]. Under conditions such as these, the intensity of radiation exposure is low but it persists for years and can impact cancer frequency for decades or generations [85].

As with high-energy, non-visible radiation that occurs on Earth (that which occurs naturally and that from medical imaging procedures and therapies), high energy space radiation, especially in this era of long-duration interplanetary travel, will likely promote premature cellular aging due to the more rapid accumulation of oxidatively-stress molecules in space travelers. Certainly, the possibility of increased colon cancer, as one example, due to high linear energy transfer (LET) radiation that occurs in space is a concern [86,87]. It is known that free radical-mediated damage accumulates more rapidly in cells/organisms exposed to LET radiation, a situation conducive to increased mutation frequency and cancer development. Having an external physical or internal physiological shield against such molecular damage in space travelers is a consideration. We have proposed the use of melatonin as one such internal safeguard to minimize the destructive actions of space irradiation [88]. The regular use of melatonin could also have other practical applications in space, e.g., promotion of restful sleep and wellbeing [89], stabilization of circadian rhythms [90,91], and the maintenance of optimal bone [92,93] and muscle health [94,95,96]. Moreover, the routine use of polychromatic lighting on the space craft that maximizes endogenous melatonin production in space travelers could be a partial solution to reducing cancer incidence as well as correcting other conditions described above [81,97,98]. Clearly, it is essential that means be found to mitigate against the harmful actions of cosmic radiation, which may be more complex than studies have revealed to date because of the unique circumstances of the space environment, e.g., microgravity [99]. We propose that melatonin would be an acceptable candidate for this purpose considering it is an endogenously-produced molecule with a virtual absence of toxicity; the latter trait is often not shared by synthetic antioxidants.

### 2.3. Other DNA Damaging Agents/Processes

**Figure 2:**
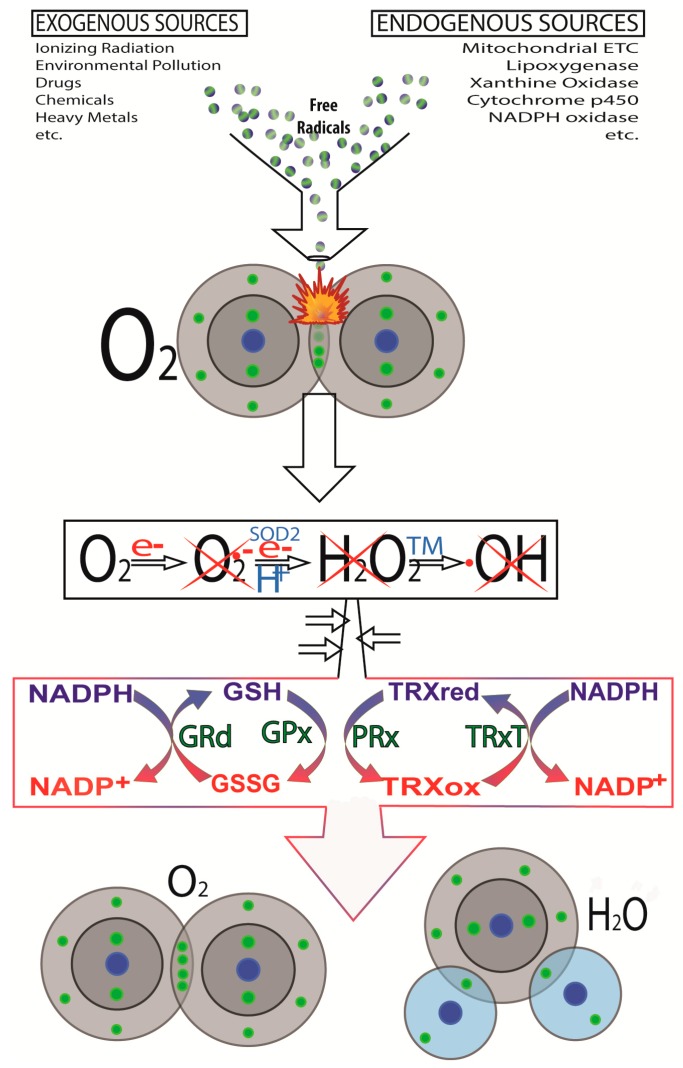
As illustrated here, there are many exogenous and endogenous factors/processes that lead to the intracellular formation of reduced oxygen derivatives. A major contributor to endogenous free radical generation is the escape of electrons (e^−^) from the respiratory complexes of the electron transport chain. This generates the O_2_•^−^ which is quickly dismutated to H_2_O_2_ and, via the Haber-Weiss/Fenton reaction, this oxygen derivative is converted to the •OH. Additionally, O_2_•^−^ produced in mitochondria can also diffuse into the cytosol through voltage-dependent anion channels (VDAC, also known as porin). The ROS marked with an X are directly scavenged by melatonin and its metabolites. Also, melatonin stimulates (↑) the activities of the enzymes which metabolize H_2_O_2_ to harmless molecules, H_2_O and O_2_. The enzymes shown to be either stimulated or protected from damage by melatonin include glutathione peroxidase (GPx), glutathione reductase (GRd) and catalase (CAT). e^−^, electron; H^+^, hydrogen atom; GSH, reduced glutathione; GSSG, oxidized glutathione; NADP^+^, nicotinamide adenine dinucleotide phosphate (reduced); NADPH, nicotinamide adenine nucleotide phosphate (oxidized); PRx, thioredoxin peroxidase; SOD2, mitochondrial superoxide dismutase; TM, transition metal; TRx, thioredoxin (oxidized/reduced); TRxP, thioredoxin reductase.

In addition to ionizing radiation, a host of other exogenous and endogenous factors contribute to genetic damage that may be a precursor to cancer initiation. As with ionizing radiation, these factors commonly induce injury by promoting the excessive generation of highly-destructive partially reduced oxygen species, i.e., ROS [100]. Some exogenous contributors to cellular DNA damage include environmental pollutants [101,102], exposure to heavy metals [103,104], toxic drugs [105,106], chemicals [107,108] (Figure 2), etc. Intracellularly, endogenous processes may also cause ROS production to the extent that it exceeds the capacity of the antioxidant defense network to fend off the damage associated with these oxygen metabolites. Most notably, misguided election flux between the respiratory complexes of the inner mitochondrial membrane causes the electrons to be misdirected allowing them to chemically reduce nearby ground state oxygen molecules to generate the O_2_•^−^ [109,110,111,112]. This occurs especially at complexes I and III. Similarly, metabolic activity, especially during enzymatic processes, cause the production of damaging ROS (Figure 2) [113]. ROS/RNS are obviously involved in the molecular damage that precedes a mutation and cancer initiation. While it is well substantiated that reactive species directly attack and damage the genome, other partially reduced species, e.g., hydroxynonenal, a product of lipid peroxidation, also has carcinogenic potential [114]. Thus, any agent or process that limits their formation or quickly neutralizes ROS/RNS would likewise lessen cancer incidence. These actions come into play especially in mitochondria since they are a major site of free radical generation.

Considering the higher concentration of the free radical scavenger melatonin in mitochondria relative to some other subcellular organelles [65], melatonin’s ability to detoxify ROS in both the intermembrane space and matrix is certainly feasible and has been documented [115]. Judging from the greater efficacy in protecting mitochondria from damage when compared to synthetically-produced, mitochondria-targeted antioxidants [116,117], melatonin itself has been classified as a mitochondria-targeted antioxidant [73,81,118] and may also have a transporter to aid in its transfer into the cell and possibly into the mitochondrial matrix [119,120], which permits it to concentrate in this organelle [65,73,81]. The superior radical scavenging activities [115,121,122,123] as well as their ability to promote antioxidant enzyme activities [75,76,78,79], allows melatonin and its metabolites to protect multiple macromolecules, including DNA, from damage thereby mitigating processes that are a forerunner of cancer.

### 2.4. Transposable Elements and DNA Damage

Genomic stability is also under the influence of transposable elements. One of these, the long interspersed element (L1), which is present in the human genome, exists as hundreds of thousands of copies with up to 100 of these loci remaining functional [124,125]. As observed in neurons, Evrony and colleagues [126] have shown that the expression of a small percentage of the fixed active L1s, and perhaps some polymorphic loci, cause 0.04–0.07 de novo insertions per normal cell [40]. A full-length functional L1 locus can induce the production of an mRNA and ORF1p and ORF2p proteins. These proteins bind to the newly-generated L1 mRNA to form a retrotranspositionally-active ribonucleoprotein (RNP). As an endogenous chaperone, Belancio and co-workers [127] have verified that ORF1p functions as a structural protein that stabilizes nucleic acids. Moreover, this group also reported that the ORF2p contains an endonuclease which can cut DNA, as well as a reverse transcriptase that is capable of synthesizing L1 cDNA in the nucleus [127]. Finally, L1 also damages genomic DNA by causing double strand breaks, which likely would have mutagenic potential.

**Figure 3:**
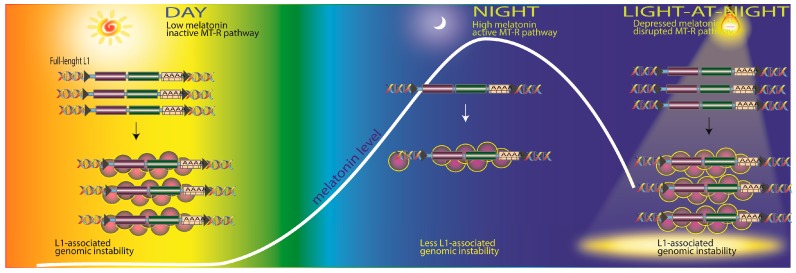
This figure illustrates the likely role of melatonin in influencing L1 expression and DNA damage. During the day (**left**) which circulating melatonin levels are at their nadir, L1 mRNA causes L1-mediated DNA damage. At night in darkness (**center**), the elevation of melatonin in the blood acts on the MT1 melatonin receptor to suppress L1 mRNA and ORF1 (open reading frame) protein. By this means, melatonin reduces L1-associated genomic stability thereby reducing the risk of cancer. Contaminating the night with light reduces melatonin levels leading to high L1 mRNA and protein expression causing L1-mediated DNA damage (**right**).

The findings of de Haro et al. [128] directly relate to this and document the important role of melatonin in influencing the expression of L1, which is commonly upregulated in human cancers. As stated above, L1 promotes genomic instability by causing double strand breaks and via insertional mutagenesis. Melatonin, by a process that requires its interaction with the MT1 membrane melatonin receptor, reduces the mobility of L1 in cultured cancer cells by downregulating L1 mRNA and the ORF1 protein. Importantly, this group also observed that melatonin-rich blood, collected from individuals at night, inhibited L1 mRNA in human cancer xenografts. Conversely, melatonin-poor blood, which was collected from individuals exposed to light at night, had no effect on L1 mRNA or ORF1 protein. From this, the group inferred that any process that reduces nighttime melatonin levels, e.g., light pollution or advanced age, would likewise enhance the likelihood of L1 expression and DNA damage (Figure 3). For additional details on the processes by which retrotransposons impact genomic stability the readers are encouraged to see reviews by Belancio et al. [129,130].

### 2.5. Melatonin and DNA Repair

Once damaged, DNA can be repaired [131,132]; these refurbishing processes also are important in reducing the risk of cancer initiation. This rebuilding of injured DNA is an on-going process and is initiated when the genome experiences any of a variety of lesions, e.g., oxidative modification, alkylation products, modified bases and single- (SSB) and double-strand breaks (DSB) [133,134], etc. The latter impairment is the most serious form of DNA damage [135]. A disfigured genome is the stimulus that initiates DNA damage response (DDR) processes [136,137]. These consist of a series of complex events that heal the DNA lesions. When the damage is highly deleterious, however, it may exceed the capacity of the DDR to repair the lesions and cell death ensues [132,138]. Elimination of seriously-damaged cells also provides protection against cancer initiation when it occurs before the cell duplicates itself [139]. While an extensive discussion of DNA repair processes is beyond the scope of this review [140,141,142], they are important in the recovery of a healthy genome and the avoidance of subsequent pathologies [137,138], e.g., cancer. Although still preliminary, there are data documenting that melatonin advances DNA repair processes [81,143,144,145]. The central role that DDR has in the maintenance of genomic stability justifies it as an important target for anticancer therapy [146].

## 3. Melatonin and Cancer Progression

There have been a plethora of published studies confirming that melatonin defers the progression of experimental cancers under both in vitro and in vivo conditions, in chemical carcinogenesis and in human cancer xenografts. Many cancer types have been studied and with multiple underlying mechanisms being proposed [40,146,147,148,149,150]. This area of research has a very long investigative history with some of the earliest studies appearing almost 40 years ago [151,152,153,154,155]. Moreover, some of the experimental findings may explain the outcome of epidemiological reports that have noted an increased cancer risk in individuals whose melatonin levels are compromised [156,157]. Among a broad cross section of experimentalists there is solid agreement that melatonin is capable of retarding cancer progression. Yet, surprisingly, its use for this purpose at the clinical level has been remarkably sparse [158,159]. This is particularly disappointing since melatonin is an endogenously-generated molecule that lacks any notable toxicity or negative side effects at virtually any dose [57,58,81]. It seems that the lack of testing, use or promotion stems from the fact that, as an inexpensive non-patentable molecule, the financial gains associated with its use would be minimal. In the US, the NIH continues to minimally support melatonin/cancer studies many of which show substantial cancer inhibition; yet these findings have not been translated to the clinical level.

Likely a consequence of a defect in oxidative metabolism in the inner mitochondrial membrane, many cancer cells exhibit an elevated production of ROS [160]. These rises in oxidative signaling have been implicated in the advancement of a variety of cancer types [161]. Moreover, ROS have been proposed to be involved in cancer initiation (as summarized above) as well as in malignant transformation (as summarized below) and in resistance to chemotherapies. In at least some cancer cells, a compensatory rise in antioxidative enzymes removes the excess ROS from the intracellular environment.

There is some evidence that prompted the speculation that higher than normal partially reduced species levels would actually promote proliferation of cancer cells. This, theoretically, would be accomplished by the ability of ROS to inactivate PI3K/AKT (serine/threonine protein kinas B) phosphatases including phosphatase and tensin homolog (PTEN) as well as protein tyrosine phosphatase 1B (PTP1B) [162,163]; these changes may expedite pro-proliferative P13K/AKT signaling [164]. Besides aiding cell proliferation, perturbations of PI3K/AKT have been linked to tumor cell survival including their resistance to some chemotherapies [162]. ROS also may be involved in advancing de novo blood vessel growth into rapidly progressing tumors [165]; angiogenesis is a necessary facet of tumor growth to supply nutrients and aid in metastasis [166].

Despite the studies delineated in the previous paragraph, by far the vast majority of data supports the notion that the high-sustained levels of ROS suppress tumor growth and function [100]. Thus, further stimulating reactive oxygen species generation (or reducing antioxidant enzyme activities) pushes cancer cells to the point of no return causing a cessation of cell proliferation and death. The actions of highly elevated ROS come into play in sensitizing cancer cells to chemotherapies [100].

**Figure 4:**
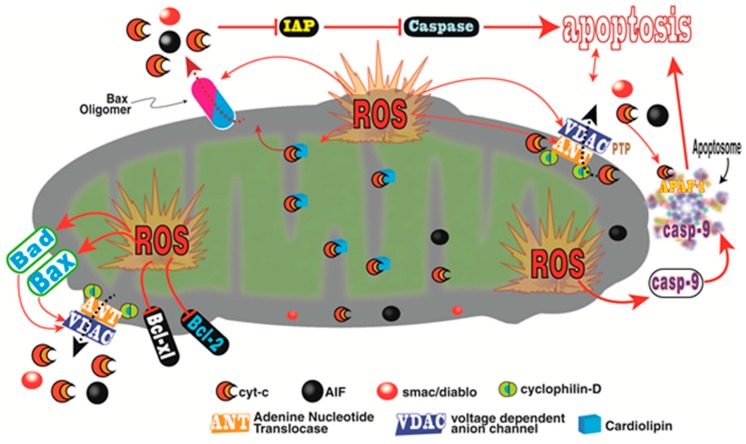
The figure illustrates some of the processes whereby melatonin may mediate apoptosis of cancer cells. In cancer cells, in contrast to normal cells, melatonin enhances reactive oxygen species (ROS) generation, which lead to cellular death via apoptosis. The text should be consulted for details.

There are a number of means by which ROS compromise tumor cell survival. O_2_•^−^ triggers the rapid release of cytochrome c from the mitochondria which contributes to the series of processes that mediate apoptosis of cancer cells; this involves a VDAC-dependent permeabilization of the mitochondria (Figure 4) [167]. In mitochondria, cytochrome c is normally complexed with cardiolipin; this complex must be breached to allow the translocation of cytochrome c into the cytosol. In the presence of H_2_O_2_, cardiolipin is oxidized thereby disassociating from cytochrome c allowing the latter to move out of the mitochondria where it continues the process of apoptosis.

Other means by which partially reduced oxygen species eliminate cancer cells is via promoting their autophagy [168,169,170], by stimulating necroptosis, i.e., type III programmed cell death [171], the recently-described ferroptosis which depends on intracellular iron concentrations but is non-apoptotic [172] and chemosensitization of tumor cells to conventional chemotherapies [173,174]. For a detailed analysis of the processes by which these signaling pathways mediate cancer cell death, the reader should consult Galadari and coworkers [100].

### 3.1. Breast Cancer

The disruption of nocturnal rise in melatonin production and secretion due to excessive light exposure during the normal dark period, i.e., light pollution, has long been known to correlate with a higher cancer risk [175,176,177,178,179,180]. Any alteration in the nocturnal rise in circulating melatonin, however, is unavoidably also accompanied by a general perturbation of circadian rhythms, which are a result of malfunction of the activity of the master biological clock, the suprachiasmatic nucleus (SCN). Given that the SCN influence the circadian organization of cellular clocks in peripheral tissues, light at night also dysregulates these cells. As a result, it is not always possible to decipher whether the reported rise in cancer frequency that is observed in humans living in an artificially nocturnally-lit environment is a result of melatonin suppression or due to circadian disturbances (chronodisruption) [181,182,183,184]. The potential involvement of disturbed biological rhythms as causative in cancer is further emphasized by the fact that the expression of two clock genes, Period 1 (Per1) and Period 2 (Per2), are known tumor suppressors in many tissues including the breast epithelium [185].

There are multiple means by which melatonin can influence breast cancer growth; some of these actions are mediated by well-known melatonin receptors while others are receptor-independent [181]. There are two membrane melatonin receptors that are members of the G protein coupled, 7-transmembrane receptor family. They are most commonly referred to as the MT1 and MT2 receptors and they are encoded by the *MTNR1A* and *MTNR1B* genes, respectively [19,186]. Both receptors are expressed in many tissues including breast epithelial cells. They are linked to a variety of different signal transduction pathways and via different G proteins [187,188,189]. The MT1 receptor in particular has been the subject of extensive investigation relative to its involvement in breast cancer [40]. Additionally, melatonin influences breast cancer via processes that do not involve the MT1/MT2 membrane receptors. Its ability to enter cells via diffusion or possibly through the glucose transporter [119] allows it to bind to the Ca^2+^-regulatory protein calmodulin [190,191]. This leads to melatonin’s ability to enhance phosphoactivation and transactivation of a number of transcription factors and nuclear binding sites that are involved in its modulation of breast cancer cell proliferation [192,193]. Melatonin also modulates RORα transcription after the indoleamine interacts with the MT1 membrane receptor [40]; this may also relate to breast cancer.

Other receptor-independent actions of melatonin that help to explain its often marked oncostatic activity includes its ability to modulate the redox status of cancer cells and possibly by altering intracellular glutathione metabolism [194]. There is also evidence that melatonin stimulates the ability of breast cancer cells to renew their shortening telomeres which would aid in the immortalization of these cells [25]. Finally, attention has recently been directed to the ability of melatonin to influence the immune microenvironment of cancer cells [195]; this could be a major means by which melatonin controls cancer cell growth. Epigenetic actions of melatonin also have been proposed to be involved in breast cancer regulation, but this field of research has not been explored extensively [196].

The results of both clinical and experimental reports have been used to justify the conclusion that melatonin is an endogenously-produced agent capable of repressing breast cancer [197,198,199,200]. This conclusion is strengthened by indirect evidence that breast cancer is more common in mid-aged/older females and in those repeatedly exposed to light at night [178,181], both of which are usually associated with lower than normal melatonin levels [201].

Essentially every aspect of melatonin’s ability to obstruct breast tumor growth has been examined. Melatonin, including at physiological concentrations (1 nM) exerts cytotoxic, anti-mitotic and pro-apoptotic actions in these cells [40,202,203]. That melatonin has antiproliferative functions have been validated in both ERα-positive and ERα-negative human breast cancer cell lines [179,204]. In the majority of these reports, melatonin acted via the MT1 membrane receptor to thwart breast cancer cell proliferation. A single report [205] claimed that both the MT1 and MT2 receptors were required for melatonin to prevent p53-mediated DNA damage. Other actions of melatonin that are likely related to the capacity of melatonin to hinder mitotic activity of mammary cancer cells includes its ability to arrest the cell cycle in the G1 phase [206], to curb aromatase activity [207], to keep in check aerobic glycolysis (Warburg effect) and to restrain the uptake of the *n*-6 fat, linoleic acid (LA), a growth factor for breast cancer cells [208].

Compared to the metabolism of normal cells, that of breast cancer cells would have to be classified as dysregulated. Nowhere is this more obvious than in the case of glucose metabolism. Many cancer cells, as described initially by Warburg [209], exhibit an uncommonly high level of glucose uptake with its metabolism by glycolysis, even when oxygen is available in abundance. This phenomenon, named the Warburg effect, is accompanied by a large rise in the utilization of glutamine which is converted eventually to α-ketoglutarate in mitochondria; this molecule then enters the tricarboxylic acid cycle (TAC). Both glucose and glutamine aid in the accelerated growth of cancer cells since they provide substrates for nucleotides via the pentose phosphate shunt (glucose) and proteins and lipids generated during the metabolism of glutamine in the TAC.

Likely the most extensively investigated model which illustrates the ability of melatonin to inhibit breast cancer growth is the one that uses human breast cancer xenografts growing in immunocompromised nude rats. In this model, tissue isolated xenografts can be carefully monitored since the single artery supplying the tumor and the single vein draining it can be cannulated. This allows for all nutrients or drugs entering the tumor and all metabolites exiting to be monitored [210].

In 2004, Blask and coworkers [17] published a seminal report which confirmed that nocturnal human blood melatonin levels are adequate to force the shutdown of human breast cancer xenografts growing in immunosuppressed rats. They perfused the tumors growing under these conditions with either daytime human blood (containing low melatonin levels) or with nighttime blood (containing elevated melatonin levels) and observed that daytime blood samples had no measureable effect on the metabolism of xenografts. In contrast, their perfusion with nighttime blood inhibited cAMP levels, linoleic acid (a growth factor for the tumors) uptake and its conversion to 13-hydroxy-octadecadienoic acid (13-HODE), a mitogenic stimulus for the tumors. Even more interestingly, if the blood donors were exposed to light at night which only partially suppressed high nocturnal blood levels (by about one-third), when perfused into the breast cancer xenografts, those samples were incapable of repressing tumor metabolism [17]. That melatonin was the molecule in the blood that was responsible for cancer inhibition was substantiated when the effects on the tumors were blocked by the use of an MT1/MT2 melatonin receptor antagonist. Moreover, adding supplemental melatonin to blood samples containing partially suppressed melatonin values restored their ability to inhibit the metabolism of the cancer transplants.

The study of Blask et al. [17] was followed by a series of reports from the same group which confirmed the capacity of melatonin to harness the metabolism and cellular proliferation in this unique xenograft model. Blask et al. [211] recently tested whether melatonin would alter aerobic glycolysis (Warburg effect) in the breast cancer xenografts. The Warburg effect is common to many tumors including breast cancer and results when the major source of ATP in cancer cells is a result of aerobic glycolysis as opposed to mitochondrial oxidative phosphorylation in normal tissues. What they found was that parameters that characterized aerobic glycolysis (glucose uptake and its conversion to lactate) and metabolic features (cAMP levels and fatty acid metabolism) were markedly impacted by the light:dark environment under which the animals possessing the xenografts were maintained. When the rats were kept under a 12:12 light:dark cycle (with darkness at night), marked circadian rhythms of tumor cAMP levels, total fatty acid levels, linoleic acid uptake and its conversion to 13-HODE were maintained. Each of these indices was suppressed at night and elevated during the day when blood melatonin levels were at their nadir. Similarly, glucose uptake, lactate production, H3-thymidine incorporation and total DNA in the tumor xenografts were high during the day and low at night, i.e., inversely correlated with the melatonin cycle [211]. When the 12:12 light:dark cycle was contaminated with low intensity light at night, a markedly different picture emerged. Thus, with the low melatonin levels throughout the 24-h period, all the tumor markers measured remained persistently in a high state of activity; these increases were correlated with accelerated tumor growth.

These findings may have important implications for the real world situation. If it only requires a modest inhibition of nighttime melatonin levels to promote breast cancer growth, females living in an artificially-lit environment (for example, in cities or night shift workers) may be prone to more rapidly growing breast tumors as already observed in epidemiological studies [177,178,181]. Likewise, since melatonin production decreases with age in most individuals, its loss may also contribute to the elevated breast cancer risk typical of older women. Finally, there may be women who suffer from endogenous hypomelatoninemia who may have an increased propensity to develop breast cancer. For the same reason, an attenuated nocturnal melatonin peak may be a biomarker to predict the likelihood of an individual to develop breast cancer, or cancer generally.

A recent review by Hill et al. [40] elegantly summarizes the reports that have comprehensively defined the role of melatonin in controlling tumor metabolism and growth in the model of transplanted xenografts in immunocompromised rats. This survey also points out the molecular mechanisms that mediate melatonin-inhibitory actions on these tumors. Since these scientists are the only group that uses this unique model, all the published reports in this area are from their laboratory.

The inhibitory actions of melatonin on experimental breast cancer obviously have been confirmed in many reports [39,40] and a variety of different actions have been proposed to explain melatonin’s antiproliferative effects in these cells. At least for MCF7 human breast cancer cells, the suppressive actions of melatonin also seem to depend, in part, on estrogen signaling; thus, the estrogen receptors α (ERα) is required. *BRAC1*, a gene that influences the susceptibility to breast cancer, functions as a ubiquitin ligase, suggesting that the ubiquitin-proteasome system may be involved in the susceptibility to breast cancer [212]. As we envision it, the ERα, apoptotic proteins and cell cycle proteins are all substrates of key ubiquitin ligases (SCF^skp2^; E6AP; SCF ^B-Trcp^) and are under the influence of melatonin. Given that the ubiquitin-proteosome system, when dysfunctional, is a risk factor for breast cancer, the model we proposed suggests the possible use of melatonin, as well as drugs that inhibit the proteasome, e.g., bortezomib, to suppress cancer [212]. For a detailed description of the hypothesis by which melatonin may utilize the ubiquitin-proteasome system to modulate breast cancer cell proliferation, the reader should consult Vriend and Reiter [212]. The combined use of melatonin and proteasome inhibitor to limit breast cancer proliferation may also have the advantage of reducing the toxicity of drugs that inhibit the proteasome [213]. Melatonin’s ability to limit the toxicity of many drugs has been repeatedly verified [214,215,216].

**Figure 5:**
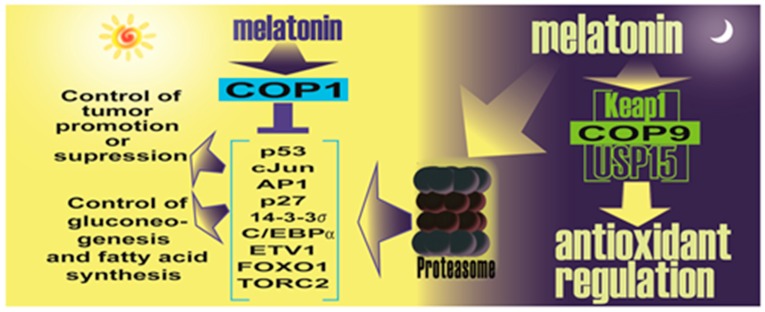
A summary of the theoretical actions of melatonin on constitutive photomorphogenic protein 1 (COP1) as it may relate to cancer. COP1, which we hypothesize is influenced by melatonin, controls the expression of multiple targets in the blue box (check color) thereby impacting tumorigenesis as well as carbohydrate and lipid metabolism. The inclusion of COP9 may potentially relate to melatonin’s antioxidant actions. The presumed direct effect of melatonin on the proteasome is also indicated.

The theory relating to melatonin and the proteasome system being involved in the anticancer actions of melatonin has recently been expanded to include a likely action on constitutive morphogenesis protein 1 (COP1) [217]. This theory also provides a context for melatonin’s antioxidant actions due to its potential interactions with the super complex formed by COP9, the oxidative stress sensor Keap1 and the deubiquitinase USP15 [217] (Figure 5).

### 3.2. Synergistic Actions of Melatonin in Breast Cancer

In 2002, we advocated the use of doxorubicin in combination with melatonin as a cancer treatment anticipating that the actions of these drugs would synergistically repress tumor growth [105], since both drugs independently block cancer cell proliferation. An additional rationale for suggesting their combined use was that melatonin may impede the toxicity of doxorubicin at other sites, e.g., the heart, without impairing the efficacy of the anthracycline at the level of the tumor. Finally, we proposed that if melatonin would reduce doxorubicin’s collateral toxicity, doxorubicin could be given at a higher treatment dose since the amount administered is normally limited by its negative actions on normal tissues.

In combination with a variety of anti-cancer treatments, melatonin invariably proved to synergize with these agents in the killing of cancer cells. For example, melatonin further stimulated apoptotic cell death promoted by arsenic trioxide; this involved an augmented production of intracellular ROS, upregulation of Redd1 expression and an activation of the p38/JNK (c-JUN-N-terminal kinase) pathway in human breast cancer cells [218]. Likewise, when combined with puromycin, melatonin synergistically had an inhibitory effect on MDA-MB 231 breast cancer cells; this included a reduction in the expression of 45S pre-rRNA while a downregulation of upstream binding factors XPO1 and IPO7, procaspase 3 and Bcl-xl [219]. When used with all-trans retinoic acid and somatostatin, melatonin was an aid to these molecules in reducing the viability and growth of MCF-7 cells [220]. Similarly, in combination with vitamin D3, melatonin amplified cell growth arrest of human breast cancer cells.

Kosar and colleagues [221] coupled doxorubicin and melatonin to specifically investigate whether the combinations would be an improved treatment for cancer. They anticipated that both drugs together would have a greater killing action on human breast cancer cells (MCF-7) than they would individually. Their particular interest was whether the transient receptor potential vanilloid 1 (TRPV1), a calcium-permeable channel that is modulated by melatonin and gated by oxidative stress, is involved in doxorubicin plus melatonin-mediated breast cancer cell death. To certify an interaction, these workers measured a variety of parameters including intracellular ROS levels, mitochondrial membrane depolarization, procaspase 9 and poly (ADP ribose) polymerase (PARP) activities, the activities of caspase 9 and caspase 3 and the degree of apoptosis. The combined treatment exaggerated these changes beyond that caused by either treatment alone. The authors concluded that melatonin supported the actions of doxorubicin due to its regulatory role on the TRPV1 channel which resulted in an increased level of apoptosis of the MCF-7 cells.

Two studies by the same group compared the ability of ionizing radiation alone or coupled with melatonin relative to cancer cell death. In both cases, melatonin sensitized cancer cells to radiation damage as exemplified by a stronger inhibitory effect on cell proliferation, cell cycle arrest and inhibition of proteins involved in DNA double-strand healing [222]. In this case, the animals were treated with melatonin in advance of the radiation therapy. The second study yielded similar results and suggested that the enhancing action of melatonin in radiation-treated cancers related to its modulation of p53 [223].

One of the concerns that was often expressed relative to the use of melatonin with anti-cancer therapies was that the indoleamine may preserve the cancer cells as it does normal tissues, i.e., it would lower the efficacy of cancer-killing drugs. As revealed by the studies summarized above, however, in each situation where melatonin was used as a complementary treatment it proved to be beneficial in that it improved the anti-cancer therapy. These findings are consistent with the context specificity of melatonin’s actions, namely that it protects normal cells from damage while further damaging cancer cells. This differential response has been seen in a number of reports [224].

### 3.3. Ovarian Cancer

Ovarian cancer, among gynecological malignancies, typically presents with a poor prognosis and is often deadly [225,226,227]. Lacking early detection methods, this cancer is frequently at an advanced stage when it is discovered. Thus, there is great urgency for new treatment strategies.

The findings of Kim et al. [228] are consistent with the potential utility of melatonin’s use as a strategy to inhibit ovarian cancer. This group reported that combining cisplatin and melatonin caused a synergistic inhibitory effect on the viability of ovarian cancer cells (SK-OV-3) while melatonin, as reported elsewhere as well, protected normal ovarian cells from cisplatin-mediated cytotoxicity. Melatonin plus cisplatin increased sub-G1 DNA content and the number of TUNEL-positive ovarian cancer cells over that caused by either treatment alone. The combination also synergistically elevated the cleavage of caspase 3 and poly-(ADP ribose) polymerase (PARP) while inhibiting the phosphorylation of extracellular signal-regulated kinase (ERK) and the dephosphorylation of 90-kDa ribosomal S6 kinase (p90RSK) and heat shock protein which are normally induced by cisplatin [228]. Based on these outcome measures, the authors concluded that melatonin may be useful as a co-treatment of ovarian cancer with cisplatin to suppress cancer progression and to ameliorate the toxic side effects of chemotherapy.

Chuffa and colleagues [229] have extensively examined the ability of melatonin to inhibit chemical-induced ovarian cancer in an ethanol-preferring rat model that was developed at their institution. These animals were selected for study since alcohol may be a co-carcinogen for ovarian cancer. The chemical-mediated tumors were induced by the injection of 7, 12-dimethyl-benz[*a*]anthracene directly under the ovarian bursa. In this model, melatonin reduced the frequency of ovarian carcinomas, sarcomas and cystic teratomas, diagnosed on the basis of histological subtype [229]. Subsequent studies confirmed that the molecular events normally associated with this type of experimental ovarian cancers were reversed by melatonin treatment [230,231,232]. Most recently, quantitative profiling of these tumors verified the broad modulatory actions of melatonin on essential signaling pathways in ovarian cancer cells [233] hinting at the possibility that melatonin should be considered as a therapeutic opportunity for women with this deadly disease. At the clinical level, melatonin has not been used as an adjuvant treatment for ovarian cancer. On the other hand, serum melatonin levels are lower in women with ovarian cancer compared to aged-match female controls. This provides feeble evidence that low endogenous melatonin may be a contributory factor to ovarian cancer.

### 3.4. Leiomyosarcoma

Using a tissue-isolated cancer growing in nude rats, Dauchy et al. [234] reported that melatonin, provided in the drinking water, markedly inhibited the growth of leiomyosarcoma tumors; this is a rare, mesenchymally-derived smooth muscle cancer. In the melatonin-fed animals the tumors regressed within 10 days while those in non-melatonin treated animals the tumors continued to grow. By calculating the amount of melatonin consumed daily, the authors felt the blood melatonin values were in the pharmacological range. Melatonin, as it does in the breast cancer xenografts described above, inhibited LA uptake and 13-HODE accumulation intracellularly. Furthermore, ERK1/2, MEK, AKT activation and 3H-thymidine uptake by the cancers were depressed as a result of melatonin consumption. These changes very likely are related to the mechanisms by which melatonin suppressed leiomyosarcoma enlargement. Since the actions of melatonin were blocked using the melatonin receptor antagonist, S20928, the authors justifiably surmised that melatonin’s actions on the cancer cells were receptor-mediated. S20928 blocks both the MT1 and MT2 membrane melatonin receptor.

The same group performed similar studies several years later. Using the same tumor type, they were infused with daytime (low melatonin) or nighttime blood (high melatonin) collected from healthy pre-menopausal females. As with their earlier study, the authors report that the infusion of nighttime blood into leiomyosarcoma xenografts suppressed all aspects of tumor growth, i.e., LA uptake, ERK1/2 and MEK levels and 13-HODE accumulation intracellularly [235]. Also, as with the first report, the endpoints measured were inhibited by the non-selective receptor antagonist, S20928. Unlike in the initial study, they [234,235] also found that melatonin suppressed aerobic glycolysis (the Warburg effect) in the smooth muscle tumors.

### 3.5. Pancreatic Cancer

Pancreatic cancer if frequently lethal since it is diagnosed late due to the absence of early symptoms and when discovered, it may be resistant to radio- and chemotherapies. The conventional chemotherapies for pancreatic cancer are gemcitabine (2,2-difluoro-2-deoxycytidine) which is frequently given in combination with 5-fluorouracil or its prodrug capecitabine [236,237]. That melatonin may improve the sensitivity of pancreatic cancer cells to chemotherapies is suggested by the findings of Uguz and coworkers [238]. They incubated rat pancreatic tumor cells (AR42J) with conventional chemotherapies (5-fluorouracil, cisplatin or doxorubicin) in the presence of melatonin and observed that this treatment caused greater mitochondrial membrane depolarization and an elevated the number of cells that underwent apoptosis relative to cells treated only with the pharmaceutical drugs. Similarly, fewer hamsters injected with N-nitrosobis (2-oxopropyl) amine (BOP) developed pancreatic cancer if they were concurrently given melatonin and those that did develop cancer survived longer [239,240]. The authors attributed the beneficial effects of melatonin to its ability to regulate the cellular redox balance. These findings indicate that melatonin may interfere with both the initiation and progression of pancreatic cancers, at least when the tumors are a consequence of BOP administration. The pro-oxidant actions of melatonin were also suggested as the means by which melatonin killed human pancreatic cells [241].

A major aspect of the use of melatonin in combination with chemotherapeutic drugs in the animals bearing pancreatic tumors is the fact that the indoleamine significantly improved normal cell function by lowering the levels of lipid peroxidation and restoring antioxidant enzyme activities [242,243]. These actions alone, regardless of melatonin’s ability to have synergistic cancer-killing activity with chemotherapies, would warrant its use in patients being treated with these devastating cancers.

Clinically, only Lissoni and collaborators [244] have used melatonin as a co-therapy for pancreatic cancer. These uncontrolled studies indicated that melatonin generally seemed to have favorable effects in terms of amplifying the chemotherapeutic efficacies of the drugs and in modulating the immunological status of the patients. On the basis of these findings alone, however, there is insufficient data to suggest the use of melatonin in clinical trials in patients with pancreatic cancer. When considered in conjunction with the outcomes of the animal studies, however, the importance of the clinical use of melatonin in pancreatic cancer patients becomes more obvious.

### 3.6. Hepatic Cancer

Hepatocellular carcinoma (HCC) is the most common hepatobiliary disease in the world and it has a high mortality rate. By the time this cancer is diagnosed, it has often advanced to the stage that available treatments display limited efficacy and, in many cases, these tumors are resistant to apoptosis so effective treatment is not possible. The resistance of HCC to apoptosis seems to involve inhibitors of apoptosis proteins (IAPs) [245,246,247] which normally prevent cell death by limiting caspase activation [248].

Based on the results of studies that preceded their investigation, Fan et al. [249] considered that possibly melatonin may overcome the resistance of HCC to apoptosis. Initially, HCC were obtained from 100 patients to examine them for the presence of several IAP members (XIAP, cIAP-1, cIAP-2, survivin and levin) using immunocytochemistry. The IAPs most frequently identified in these specimens were XIAP, cIAP-1, cIAP-2 and survivin. To determine the effect of melatonin on these proteins, two human hepatoma cell lines were used, Hep G2 and SMMC-7721. The first obvious effect of melatonin was that the indole significantly inhibited the growth of both hepatoma cell lines. This change was associated with the induction of apoptosis accompanied by downregulation of XIAP and survivin. Melatonin also lowered the expression of COX-2 and reduced AKT activation in both Hep G2 and SMMC-7721 cells. With the aid of a selective inhibitor of COX-2, LY 294002, Fan and colleagues [249] documented that melatonin-mediated apoptosis targeted the COX-2/PI3K/AKT pathway resulting in the suppression of the IAPs, survivin and XIAP. The authors suggest that the ability of melatonin to reverse apoptosis resistance in hepatoma cells may make this agent a valuable therapy for HCC. The inhibitory action of melatonin on hepatic cancer cell growth may have been even more dramatic had it been given in combination with conventional therapies for this condition.

With the assumption that using melatonin with a conventional drug would kill additional hepatoma cells, Lin and coworkers [250] combined sorafenib, the only pharmaceutical agent approved for the treatment of HCC, and melatonin. Each drug independently and in a dose-dependent manner reduced the proliferation of HuH7 hepatoma cells. When used as a co-treatment, melatonin plus sorafenib synergistically enhanced the growth inhibitory effect on the hepatoma cells including a decrease in their clonogenicity and an elevated apoptosis. These changes were correlated with activation of caspase 3 and of the JNK/c-Jun pathway. The inhibitor of c-Jun phosphorylation, SP600125, prevented the enhanced apoptosis induced by the combined treatment. The JNK/c-Jun pathway can be activated by multiple agents/processes, one of which is ROS. Thus, melatonin may have improved the cancer cell killing ability during the combined treatment by functioning as a pro-oxidant, which it often does in cancer cells [224,251]. There are, however, other potential explanations as well [252].

Considering how common and how serious HCC is worldwide, treatment options are a rather unmet need. Based on the high efficacy of melatonin in the inhibition of experimental hepatocellular carcinoma coupled with the essential absence of adverse events when used by humans, it should be tried as a co-treatment in patients with this catastrophic disease.

### 3.7. Colorectal Cancer

Colorectal cancer is of great concern since it is a common malignancy and is the second most frequent cause of cancer deaths worldwide [226]. This cancer is generally considered to be related to diet, namely, a diet consisting of high amounts of meat and low in fiber-rich vegetables and fruit. Wei and colleagues [253] examined the ability of melatonin to promote apoptosis of colon cancer cells and attempted to define the mechanisms by which melatonin achieved this in a human colorectal cell line (LoVo). They found that melatonin’s ability to stimulate apoptosis of these cancer cells was associated with dephosphorylation and inhibition of the import of histone deacetylase (HDAC4) into the nucleus. Melatonin-mediated apoptosis was prevented when cells were treated with HDAC4-specific siRNA. Wei et al. [253] also observed that constitutively active Ca^2+^/calmodulin-dependent protein kinase II α (CaMKIIα) reduced the nuclear translocation of HDAC4 and limited program cell death of the colorectal cancer cells. This study not only documents the ability of melatonin to enhance the destruction of colon cancer cells, as shown in an earlier study as well [254], but also provides some information related to the possible mechanism involved. Since this cancer may be influenced by the diet, the findings are of special interest since fiber-rich vegetables and fruits, which are not a major part of the western diet, normally contain significantly higher amounts of melatonin than do animal products [255,256,257].

Leon et al. [258] also investigated the role of melatonin in controlling the growth of colorectal cancer cells. Using Caco-2 and TP4 colon cancer cell lines, both of human origin, they explored endothelial-1 (ET-1) as a factor influencing colorectal cell growth and invasion. ET-1, a peptide secreted by many solid tumors, functions as a survival factor for the cancer cells by stimulating cell proliferation, blood vessel ingrowth and repressing apoptosis. When added to the culture medium, melatonin inhibited *edn-1* mRNA expression (which is the initial step in ET-1 synthesis) and the release of ET-1 from the colorectal cancer cells. The reduction in *edn-1*expression was related to an inactivation of FoxO and NF-κB transcription. The authors took these findings as evidence that melatonin, which is normally synthesized in the gastrointestinal tract, may be involved in inhibiting cancer of the colon by influencing ET-1 production.

### 3.8. Lung Cancer

We recently proposed that melatonin should be tried as a therapy for non-small-cell lung cancer (NSCLC) [120]. This is a common cancer with a poor 5-year survival rate of 10%–20% [259]. Surgical removal of the tumor and chemoradiotherapy are the most common treatment paradigms but they have limited effectiveness. Moreover, the latter therapy has serious side effects on non-cancer cells which also curtail its use [260]. Thus, there is an aggressive search for non-toxic agents that may be a direct treatment for NSCLC or as a co-adjuvant that would reduce the undesirable consequences of the conventional treatments.

Melatonin, as noted herein, inhibits a variety of cancer types which includes lung cancer in mice induced by urethane [261,262]. Proliferating-cell nuclear antigen (PCNA) is considered a molecule marker for proliferation with PCNA inhibition being considered as a reasonable anticancer strategy [263]. Fan et al. [264] have shown that melatonin downregulates PCNA expression and increases death of A549 and PC9 lung cancer cells. There is also evidence that melatonin may reduce the metastasis of NSCLC [265]. The reduction of the metastatic potential of lung cancer cells was associated with lowered expression of myosin light chain kinase (MLCK) and osteopontin (OPN) and the phosphorylation of myosin light chain (MLC) along with the upregulation of expression of occludin, which is involved the c-jun-N-terminal kinesis (JNK) pathway. In light of these findings, Fan and coworkers [264] suggested melatonin as a treatment for NSCLC and also as a co-treatment with chemo- or radio-therapy. The co-treatment suggestion was made with the hope of reducing the noxious side effects of these conventional drugs, thereby improving the wellbeing of the patients.

### 3.9. Bone Cancer

Melatonin was shown to inhibit MG-63 osteosarcoma cell proliferation but mechanistically this action remains unexplained [266,267]. Considering other studies showing that melatonin inhibition of cancer cell growth involves mitogen-activated kinases and AKT signaling, Liu et al. [268] examined changes in these signaling pathways in an attempt to determine if they also relate to the mechanisms by which melatonin interferes with the proliferation of osteosarcoma cells. In human MG-63 osteosarcoma cells, melatonin significantly inhibited the phophoactivation of ERK1/2 but was without effect on either p38, JNK or AKT. This action of melatonin was duplicated when the cells were treated with a selective inhibitor, PD98059, of MEK. Likewise, when MG-63 cells were incubated with a combination of melatonin and PD98059, it highly effectively blocked ERK1/2 activation. Thus, ERK1/2 inhibition is likely involved in the process by which melatonin arrests MG-63 osteosarcoma cells in the G1 and G2/M phases of the cell cycle [268]. In contrast, since p38, JNK and AKT were not changed by melatonin treatment, it was concluded these pathways are not involved.

### 3.10. Glioblastoma

Glioblastoma-like stem cells were isolated from glioblastoma multiforme tumors of patients for the purpose of exploring the possible role of melatonin in modulating their growth [269]. These primary cultures were treated with 1 mol/L melatonin, a concentration previously shown to interfere with malignant progression of these cells [270,271]. Melatonin had a marked inhibitory effect on cancer cell proliferation and the interaction of EZH2-STAT3 in these cells along with the phosphorylation of this complex also being impaired as a result of melatonin treatment. Clearly, melatonin had inhibitory actions on glioblastoma stem cells in culture; if these same interactions occur in multiforme glioblastoma tumors in vivo, melatonin may prove to be useful in targeting this cancer type. The authors did not test whether melatonin’s capacity in altering growth of glioblastoma-like stem cells involved the classic membrane receptors for the indoleamine. If applicable to the clinical level, the findings of Chen et al. [269] would be highly important given that glioblastoma multiforme is the most common of the glial tumors and is highly aggressive.

### 3.11. Leukemia

That melatonin modulated the induction of apoptosis in hyperthermia-treated human leukemia cells (0937) was verified by Quintana and coworkers [272]. Hyperthermia has emerged as a common approach to aid in the killing of cancer cells; it is usually combined with radio- or chemotherapy [273,274]. In the current study, melatonin, which by itself is an anti-cancer agent, when combined with hyperthermia (43 °C) enhanced the apoptotic responses of human acute myeloid leukemia cells. Hyperthermia triggered apoptosis via mechanisms involving caspase 8, hydrolysis of Bid, release of cytochrome C from mitochondria followed by the activation of caspase 9 and caspase 3 [272]. When melatonin was added to the incubation medium, it greatly amplified hyperthermia-mediated cancer cell death by exaggerating the intrinsic apoptotic pathway. While melatonin is well recognized for its low toxicity under euthermia body temperatures, its effects during whole body induced hyperthermia, which is used to treat multiple metastatic sites, has yet to be tested.

### 3.12. Prostate Cancer

Blood melatonin levels are inversely related to the progression of prostate cancer [275]. Patients with elevated concentrations of the major urinary melatonin metabolite, 6-hydroxymelatonin sulfate, are significantly less likely to have an advanced stage of the disease [276], and the oral intake of the pineal indoleamine slowed biochemical relapse of the androgen-independent prostate tumor in a clinical case [277]. These findings indicate a central role of melatonin in repressing prostate cancer progression, and further suggest the promise of melatonin as an anticancer agent. The therapeutic potential of this pineal secretory product is attributed to its diverse physiological functions that restrain the aggressive behaviors of prostate cancer cells [278].

Abundant experimental evidence demonstrates that melatonin exerts an anti-proliferative effect on both and rogen-sensitive or hormone-refractory human cancer cells [279,280], and that the in vivo treatment with melatonin reduces the size of xenograft tumors in intact or castrated animal models [281,282]. The growth inhibitory action of melatonin involves the induction of either apoptosis [283] or cell cycle arrest [284]. In addition, melatonin administration was shown to limit microvessel density and impair the vascularization in LNCaP xenograft models [285,286,287]. These observations suggest that melatonin interferes with the processes of angiogenesis in prostate tumors, which is a major factor driving cancer progression [288]. Melatonin also controls the generation of reactive oxygen species (ROS) and significantly increases the total content of glutathione in prostate cancer cells [289,290]. Due to its actions in redox regulation, melatonin modulates intracellular oxidant stress and thereby determines malignant progression of the disease. Additionally, the indoleamine influences the production of cytokines and chemokines by human prostate cancer cells [291] and in patients with advanced prostate tumors [292]. These findings suggest either anti-inflammatory or immunostimulatory functions of melatonin in augmenting the host defense against cancer progression. Interestingly, melatonin has been shown to promote the neuroendocrine (NE) differentiation of prostate cancer cells [293,294]. In general, NE cellular transdifferentiation supports the aggressive outgrowth of existing prostate tumors in an androgen-independent manner, and facilitates the development of resistance to anticancer therapy [295]. Thus, the acquisition of the NE phenotype after melatonin treatment seems to be contradictory to the oncostatic properties of the indole. Melatonin induced the appearance of long dendritic-like extensions and smaller, rounded cell bodies, a morphological change similar to that occurring when prostate cancer cells undergo NE differentiation. However, distinct molecular features, including NE markers and gene expression patterns were not detected in response to melatonin compared with regular differentiation signals such as androgen withdrawal [294]. Melatonin administration prolonged the life span of TRAMP (transgenic adenocarcinoma of the mouse prostate) mice, which is a classical model of NE carcinoma [296], and effectively blocked tumor growth [294]. Thus, how exactly melatonin triggers NE differentiation of prostate cancer cells, and whether this process contributes to tumor suppressive activity of the indoleamine is still a matter of debate.

The wide spectrum of biological effects elicited by melatonin underscores the diverse mechanisms of its action to counteract prostate cancer progression. It is now generally accepted that melatonin exerts its anticancer function either through an interaction with its specific receptors or in a receptor-independent manner. Two forms of protein receptors (MT1 and MT2) have been identified in humans, with different binding affinity for melatonin. They are both G-protein-coupled receptors and promote a cascade of downstream signaling upon an interaction with the indole.

The discovery of melatonin receptors on prostate cancer cells, especially MT1, significantly improved understanding of the molecular mechanisms underlying melatonin’s antitumor activity in advanced prostate cancer. Expression of MT1 was detected in human prostate cancer cell lines [279,280,297], xenograft tissues [281] as well as in patient samples [277]. When androgen-responsive prostate cancer LNCaP cells were treated with the analog of melatonin, 2-[^125^I]-melatonin, which is also a high-affinity MT1 agonist, cell proliferation was dramatically inhibited [277,280]. Furthermore, the effects of 2-iodomelatonin were specifically depressed by luzindole, a non-selective antagonist of both MT1 and MT2, but were not influenced by the selective MT2 antagonist 4-phenyl-2-propionamidotetraline (4-P-PDOT) [298]. The accumulated evidence strongly supports that the involvement of the melatonin receptor, MT1 in particular, in mediating the inhibitory effects of melatonin on the growth of prostate cancer cells. MT1 acts on multiple signaling pathways, for example, it activates protein kinases A and C (PKA and PKC) in parallel, which leads to the transcriptional upregulation of cell cycle inhibitor p27^Kip1^ [297,299]. At the same time, the melatonin-MT1 axis represses the growth-stimulating effects of epidermal growth factor (EGF) by attenuating EGF-induced expression of cyclin D1, and thereby blocking cell cycle progression though the G1 phase [282].

One of the most important interactions the activated MT1 has relates to androgen receptor (AR) signaling. It is well known that the AR-regulated transcriptional program plays a vital role in driving progression of prostate cancer [300]. Melatonin treatment does not modulate AR protein levels, binding of AR with its ligand androgen, or recruitment of AR to its regulatory elements on chromatin. Rather, MT1 causes the translocation of AR from nucleus to cytoplasm, which is dependent on the activation of PKC signaling, and thus decreases the transcriptional activity of the nuclear receptor [301,302,303]. These varied actions that melatonin receptors perform collectively account for the anti-proliferative activity of melatonin and retard the development of prostate tumors to a more advanced stages.

There is still controversy, however, regarding the exact role of the melatonin receptors in melatonin-mediated protection against prostate cancer progression. Although identified on AR-positive LNCaP cells, binding sites for 2-[^125^I]-melatonin were undetectable on the membrane of AR-negative prostate cancer cells such as PC3 and DU145 [279,282]. These findings suggested low levels or even absence of melatonin receptors, and thus the biological effects of melatonin in these cell models were possibly not mediated by the activity of specific receptors. Interestingly, MT1-dependent mechanisms of melatonin action were defective in AR-null, but not AR-intact, prostate cancer cells, which may further imply a functional interplay between melatonin-MT1 axis and AR signaling. Surprisingly in a radioreceptor assay, binding sites for 2-[^125^I]-melatonin, were found in nuclear extracts of DU145 cells [279], implicating the presence of melatonin receptors in the nuclear compartment. Additional evidence is required to confirm this observation, and the function of this nucleus-localized melatonin receptor in prostate cancer progression needs further exploration.

**Figure 6:**
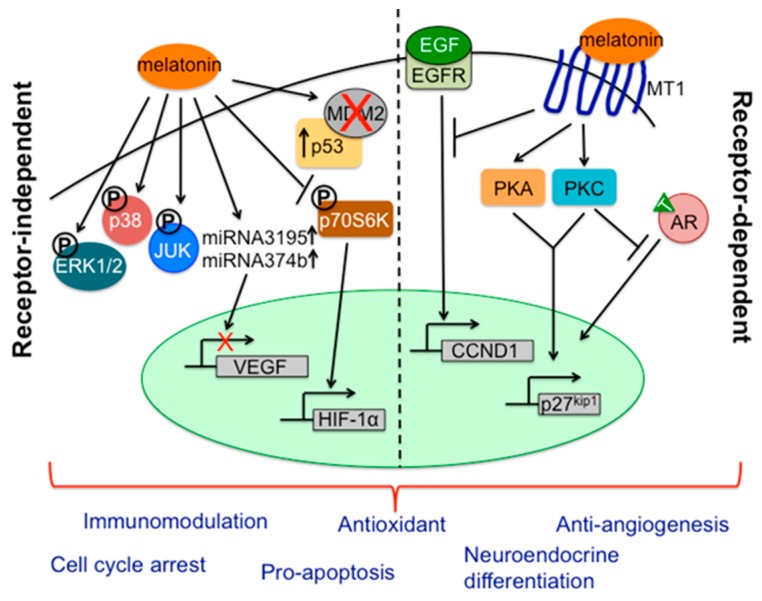
Mechanisms of melatonin’s oncostatic action during prostate cancer progression; these are either independent of the functions of melatonin receptors (**left**) or mediated via specific interaction of the indoleamine with its receptors (**right**). Multiple signaling cascades are induced or inactivated in prostate cancer cells upon melatonin treatment, which results in a variety of biological effects of the pineal secretory product. See text for additional details. T, testosterone; P, phosphorylation.

Mechanisms with no involvement of the protein receptors for melatonin have also been demonstrated in prostate cancer. In these scenarios, the indole directly triggers a series of signaling cascades that ultimately hinder the progressive growth of advanced prostate cancer cells. For example, p38 kinase and c-JUN N-terminal kinase (JNK) were both activated and directly contributed to melatonin-induced apoptotic death in LNCaP cells [304]. P53 was also reported to participate in the pro-apoptotic action of melatonin in prostate cancer cells, as the indoleamine repressed the levels of negative regulators of p53 including MDM2 and Sirt1 [224,283]. NE differentiation triggered by chronic treatment with melatonin was independent of melatonin membrane receptors or the PKA activity [293], but required the persistent activation of ERK/MAPK pathway [294]. Moreover, melatonin attenuated the levels of HIF-1α protein by abrogation of p70S6K/RPS6/eIF4B pathway [305], which is responsible for the translation of HIF-1α gene [306]. Apart from signal transduction, melatonin enhanced the expression miRNA3195 and miRNA374b, an epigenetic mechanism to silence vascular endothelial growth factor (VEGF) and HIF-1α genes [287]. Hence, melatonin restrained the abundance of angiogenic factors, provoking anti-angiogenic and antioxidant effects to interfere with prostate cancer progression [305]. Taken together, these downstream signaling events lead to a plethora of biological effects of melatonin, which likely contribute to the oncostatic properties of melatonin in advanced prostate cancer (Figure 6).

## 4. Melatonin: Causing Treatment Resistant Cancer to Become Sensitive to Treatment

The resistance of tumors to endocrine-related therapies often complicates their successful treatment. As an example, breast cancer is frequently resistant to anti-estrogen treatments. In vitro studies have shown that ERα+ MCF-7 human breast cancer cells become responsive to anti-estrogen treatment if melatonin is added to the culture medium [307]. Additionally, in situations where darkness is contaminated with light (light pollution) or in aged individuals (where melatonin levels are low) the resistance of cancer to drugs may be a consequence.

**Figure 7:**
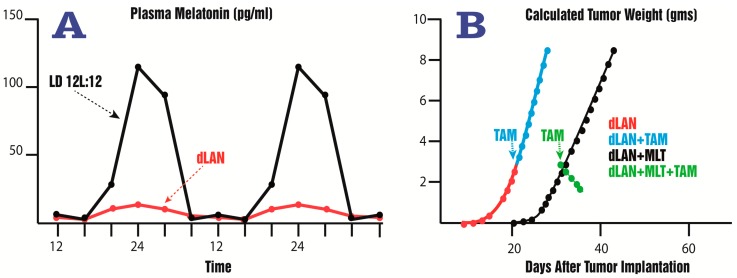
Differential responses of subcutaneously growing human breast tumors to daily intraperitoneally-injected tamoxifen (TAM) alone or concurrently with melatonin (MLT, given in the drinking water) in rats exposed to dim light at night (dLAN). The light:dark cycle was 12:12. dLAN suppressed the nocturnal rise (**A**) in endogenous melatonin which was restored by the addition of melatonin to the drinking water. dLAN (red dots and line) advanced the latency to recognizable tumor growth (**B**) compared to that in rats exposed to 12:12 where the night was dark (black dots and line). Also, TAM administered to rats exposed to dLAN failed to inhibit tumor growth (blue dots and lines), i.e., they were resistant to TAM. By comparison, when TAM treatment was instituted in rats that were given melatonin, tumor enlargement was inhibited (green dots and lines), i.e., they became sensitive to TAM. The speculated mechanisms to explain the elevated sensitivity of the tumors to TAM are multiple and are summarized in the text. Figure drawn from the data published by Dauchy et al. [308].

Based on observations such as these, Dauchy and colleagues [308] tested whether a dampened circadian melatonin rhythm contributed to tumor resistance to a conventional therapy, in this case tamoxifen (TAM), in an animal model. Athymic nude rats bearing MCF ERα+ breast cancer cells implanted subcutaneously were housed under a light:dark cycle of 12:12 where the night was either dark or contaminated with low intensity light (referred to as dim light exposure at night or dLAN), which substantially restrained the nocturnal increase in circulating melatonin levels. As shown in earlier studies [309,310], dLAN advanced the latency to onset of tumors compared to those in rats exposed to nighttime darkness. When the dLAN exposed animals were given TAM, the tumors were resistant to the drug and continued to enlarge. Conversely, in rats kept under daytime light and 12-h darkness at night, TAM treatment quickly caused tumor growth to cease, i.e., they were not resistant to TAM treatment (Figure 7). Hence, the nocturnal suppression of melatonin due to dLAN was likely consequential in decreasing the sensitivity of the tumors to TAM [308]. Circadian disturbances could not, however, be completely dismissed as a contributing factor in TAM resistance since, while the circadian system continued to operate, its function was severely compromised.

In addition to monitoring cancer enlargement, at the conclusion of their study Dauchy et al. [308] also measured a variety of physiological, metabolic and molecular parameters of these tumors. While the results did not identify the specific mechanisms that account for TAM resistance (an increased sensitivity after melatonin treatment), it seems likely that these processes are complex and involve yet to be identified signaling pathways as well as transcriptional and metabolic events. Melatonin treatment did, however, have metabolic and molecular consequences in the tumors that may have changed their sensitivity to TAM. For example, it suppressed aerobic glycolysis (the Warburg effect), the uptake of LA and its conversion to the mitogenic agent, 13-HODE. Melatonin also greatly attenuated the expression of total and phosphorylated ERK1/2, AKT, SRC, cAMP, STAT3, FAK, LPEB, and NK-κB. Some of these processes have been previously implicated in tumor resistance to TAM [311,312]. STAT3 may deserve special attention in this situation since it is a point of convergence of a number of cancer-inhibiting signals and it is involved in cell proliferation, metabolic activity and survival of many tumors [311]. STAT3, which is inhibited by melatonin, promotes the expression of genes important for tumor growth, i.e., Myc and cyclin D1 [313]. Certainly, the work of Dauchy et al. [308] comes closest to explaining the molecular processes by which melatonin modulates the sensitivity of human breast cancer to TAM specifically and possibly to chemotherapies more generally.

This group feels that the suppressed melatonin due to dLAN is the major contributor to the loss of sensitivity of these tumors to TAM. If so, this has obvious implications for the treatment of human cancers since, in cities throughout the world, contamination of the daily dark period (light pollution) is rampant. In fact, epidemiological studies have shown that certain types of cancers are more common in an urban environment, in which contamination of the night with artificial light is common, than in the rural environment, where light pollution is reduced [314]. Other cancer types that were shown to be resistant to various conventional therapies but become sensitive when exposed to melatonin are summarized in Table 1.

## 5. Role of Melatonin in Cancer Metastasis

Cancer metastasis, responsible for most deaths due to malignancies, is a multistage process that requires cancer cells to escape from the primary location, survive in the circulation, and grow at distant sites [318]. Due to the broad range of melatonin’s functionality, efforts to understand the oncostatic role of melatonin have recently shifted toward the process of tumor metastases. Mounting evidence has linked melatonin’s actions to many cellular and organismic mechanisms of metastasis rendered by both cancer and non-neoplastic cells within the tumor microenvironment [319]. These include regulation of cell-cell and cell-matrix interaction, extracellular matrix (ECM) remodeling by matrix metalloproteinases (MMPs), cytoskeleton reorganization, epithelial-mesenchymal transition (EMT), and angiogenesis.

A characterized feature of disseminated tumor cells is the substantial flexibility in their adhesive interactions with neighboring cells or ECM elements. As cancer cells undergo invasion, the disruption of cell-cell junctions permits cells to disassociate from the primary tumor mass, and changes in cell-matrix interaction allow the cells to penetrate into the adjacent stroma [320]. A variety of compositionally and functionally distinct epithelial surface structures, such as tight junctions (TJs), adherens junctions (AJs), gap junctions (GJs), desmosomes and hemidesmosomes are responsible for maintaining cell polarization, collectively preserving the cellular architecture of epithelium. Of these, it has been shown that melatonin exerted an inhibitory effect on cancer invasion via modulating the expression of cell adhesion molecules associated with TJ and AJ. A notable example is that melatonin has been shown to convert a human breast cancer cell line, MCF-7, to a reduced invasive phenotype by increasing expression of E-cadherin, a prototypical member of the type-1 classical cadherins found at AJs [321].

Loss of E-cadherin in cancer cells is considered a hallmark alteration of cell junction in the early step of metastasis [322]. In addition to affecting the intercellular interactions, loss of E-cadherin leads to metastatic dissemination by the regulation of numerous signal pathways and induction of many transcription factors through its intracellular binding partner, β-catenin [323,324]. Recently, in a study of gastric cancer dissemination, melatonin’s action on upregulation of E-cadherin was shown to be controlled by interruption of NF-κB interaction with C/EBPβ [325]. Another cell-cell junction implicated in negative effects of melatonin on tumor spread and metastasis is TJs. As a control for the paracellular diffusion of ions and various molecules, TJs occupy pivotal roles in the maintenance of cell integrity. While the cohesion of the TJ architecture is perturbed by altered expression and/or distribution of TJ proteins, cancer cells can become highly mobile and invasive [326]. Elevated levels of occludin, a transmembrane protein present in TJs, have been observed as melatonin effectively counteracted the migration of a human lung cancer cell line, A549 [327]. Occludin was shown to regulate membrane-localized activation of PI3K and leading-edge localization of polarity proteins, aPKC-Par3 and PATJ, to promote directional migration of epithelial cells [328].

Exquisite expression of genes encoding the adhesion molecules involved in cell-to-ECM interaction has been linked to melatonin’s actions on invading cancer cells. A noticeable instance is the integrin receptor, a heterodimeric glycoprotein family that connects cues from extracellular milieu to intracellular signaling apparatus [329]. It has been reported that expression of α_v_β_3_ integrin was dampened while melatonin exerted its inhibitory effects on glioma cell invasion under hypoxic conditions [330]. The expression level of α_v_ integrin was demonstrated to be not only correlated with but also functionally implicated in retaining a highly invasive status in prostate and throat cancers [331,332]. In contrast, the migratory response of breast cancer cells was repressed by melatonin through the induction of β_1_ integrin [321]. These results reflect that melatonin mediates a well-coordinated interplay between cell-cell and cell-matrix adhesion, restraining the neoplastic cells from escaping the primary tumor mass and invading the neighboring stroma.

In addition to a local microenvironment that supports the primary tumor cells to migrate, metastasis is also in need of a pre-metastatic niche for circulating tumor cells to lodge and colonize at distant sites [333]. The major component of such “foreign soil” is the ECM, a highly dynamic structure that is present in all organs and constantly being remodeled. The most significant enzymes responsible for ECM remodeling are MMPs [334,335]. ECM provides not only essential physical scaffolding for adhesion molecules to attach but also serves as a ligand reservoir by sequestering various growth factors such as basic fibroblast growth factor (bFGF) and vascular endothelial growth factor (VEGF) [336]. Degradation of ECM by MMPs releases these crucial biochemical cues, which in turn boost cancer dissemination by inducing angiogenesis and lymphangiogenesis [337,338,339]. Melatonin has been shown to modulate the function and expression level of MMPs [334]. It is reported that melatonin exhibited a repressive effect on the catalytic activity of MMP-9 through directly docking into its active site in a gastric adenocarcinoma cell line [340]. In addition, melatonin-mediated downregulation of MMP-9 was found to be attributed to an epigenetic or NF-κB-dependent transcriptional regulation in investigations of oral cancer cell migration [341], kidney cancer metastasis [342] and nasopharyngeal carcinoma cell migration [335], respectively. These findings reveal a prevailing role for melatonin to counteract tumor metastasis via MMP-9-mediated ECM remodeling.

As stated above, the exquisite crosstalk between cell-cell and cell-matrix interactions controls the flexibility of cancer cells that enables them to receive extracellular cues, from either the ECM itself or from ECM-interacting molecules. These external signals govern the organization of cytoskeletal proteins, which are central to direct cell movement. An inhibitory effect of melatonin on breast cancer cell migration and metastasis through ROCK-regulated (Rho-associated protein kinase) rearrangement of microfilament and microtubules has been verified. In addition to hindering the enzymatic activity of ROCK kinase, melatonin consistently curbed cancer invasion by attenuating the expression of several key regulators of the cytoskeleton, such as ROCK-1 [343,344] and MLCK [326]; this pinpoints a capacity of melatonin in cytoskeleton reorganization via orchestrating various signaling molecules during tumor metastasis.

Accumulating evidence has suggested that manipulation of epithelial-mesenchymal transition (EMT), a process whereby epithelial cells shift toward the mesenchymal phenotype and become highly mobile [345], significantly influences cancer development and metastasis [345,346,347]. Such a switch in cell polarity, morphology, and behavior is controlled by a myriad of transcriptional and signaling events triggered by numerous external stimuli [174]. Signaling cascades, such as NF-κB, Wnt, Notch, Hedgehog, AP-1, and growth factor [348,349,350], as well as their downstream transcription factors (e.g., Snail, Slug, Twist, and Zeb) [346] have been shown to be regulated in metastatic cells that are undergoing EMT. It has been documented that melatonin hinders EMT and cancer cell dissemination via interference of the NF-κB signaling [325].

Another major regulator of EMT that has been involved in melatonin’s actions is the Wnt/β-catenin pathway. Due to its capacity to bind to E-cadherin, β-catenin represents a central constituent of the adherens junctions [351]. As EMT proceeds, downregulation of E-cadherin liberates β-catenin from the junctional structure, allowing the translocation of β-catenin into the nuclei where β-catenin acts as a transcription factor to turn on the expression of Wnt target genes, including Snail and Slug. The surplus of cytoplasmic β-catenin is phosphorylated and targeted to the proteasome for degradation by a destruction complex made up of glycogen synthase kinase 3 β (GSK3β), axin, adenomatous polyposis coli (APC), and casein kinase 1, as the Wnt signal is shut off. It is documented that melatonin activates GSK3β by interfering with Akt phosphorylation to promote β-catenin breakdown and impede the EMT in a xenograft model of breast cancer metastasis [352], suggesting a circadian control of EMT in the spread of malignancies via regulation of GSK3β. Additionally, an oncogenic growth factor signal, the HER-2 (human epidermal growth factor receptor-2)/MAPK/Erk pathway, was recently reported to be involved in melatonin-mediated repression of EMT and late-staged metastasis in breast cancer cells via Rsk2 [353]. This result opens a possibility for a supportive use of melatonin in the treatment of HER2-positive breast cancer.

During cancer development and progression, extensive capillary structures develop to provide not only oxygen and nutrients but also a path for neoplastic cells to escape from the primary site. The process of angiogenesis, defined as the formation of new blood vessels from the pre-existing vasculature requires intricate interactions between endothelial cells and the microenvironment [354]. Several key signaling pathways, including but not limited to, growth factors, integrins, cell surface receptors, cytokines, chemokines, lipids, and the ECM, are recognized to be crucial for angiogenesis [355]. Among these, VEGF is a strong pro-angiogenic growth factor that plays a key role in tumor angiogenesis and metastasis [356] and has emerged as a therapeutic target of cancer therapy [357]. The impact of melatonin on inhibition of angiogenesis was shown to be related to a reduction in VEGF secretion in patients with progressive tumors [36]. Such an inhibitory effect can be explained by the observations from numerous cancer studies that melatonin promoted the turnover of hypoxia-inducible factor-1α (HIF-1α), a transcription factor known to leverage gene expression of VEGF [271,287,358,359].

**Figure 8:**
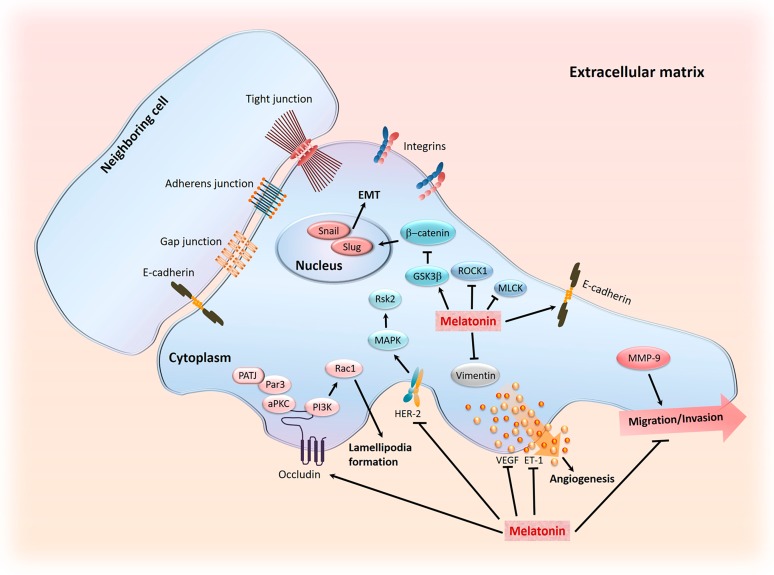
Schematic representation of the multiple mechanisms involved in melatonin-mediated inhibition of cancer metastasis. EMT: Epithelial-mesenchymal transition; ET1: Endothelin-1; GSK3β: Glycogen synthase kinase 3β; HER-2: Human epidermal growth factor receptor-2; MAPK: Mitogen-activated protein kinase; MLCK: Myosin light-chain kinase; MMP-9: Matrix metalloproteinase-9; ROCK1: Rho-associated protein kinase 1; VEGF: Vascular endothelial growth factor.

Melatonin was also shown to attenuate the expression and release of endothelin-1 (ET-1) in colon cancer cells through the inactivation of FoxO1 and NF-κB [258]. ET-1 functions as a vasoconstrictor that influences multiple steps of tumor angiogenesis [360]. These findings highlight an additional role of melatonin in tumor metastasis by counteracting the angiogenic responses. These and other actions of melatonin in determining cancer cell invasion and metastasis are summarized in Figure 8.

## 6. Concluding Remarks and Perspectives

**Figure 9:**
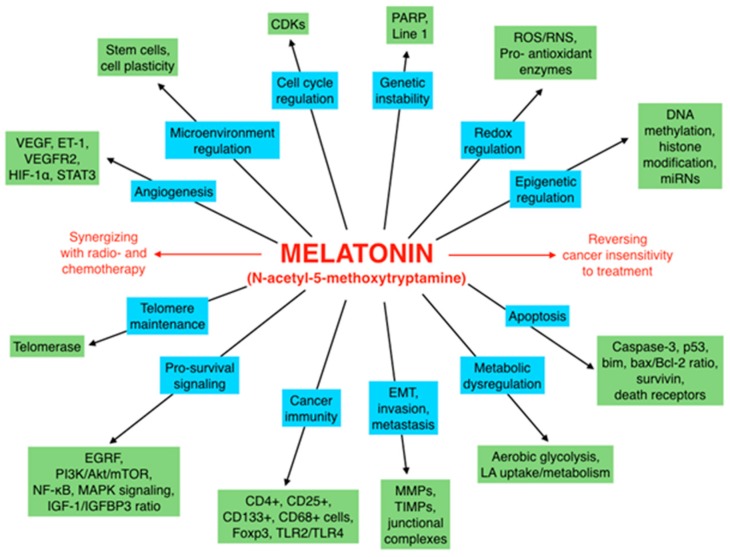
The multiple means that have been proposed by which melatonin may interfere with the growth of experimental tumors are listed. The blue boxes identify the process that are impacted by melatonin while the green boxes list the potential mechanisms involved. The information in red mentions the synergistic actions of melatonin with radio- or chemotherapies (**left**) and on the **right** it is noted that some cancers resistant to therapy can be made sensitive by treatment with melatonin.

The amount of published data substantiating melatonin’s oncostatic activity on experimental cancers is vast. What is somewhat baffling, however, is the widely-varied mechanisms that have been proposed to explain the processes by which melatonin accomplishes these activities (Figure 9). It seems there are as many proffered mechanisms as there are tumor types that have been reported to be constrained by the indoleamine. For example, the following actions of melatonin have been tentatively proposed to explain its anti-cancer activity: anti-angiogenesis, modulation of signal transduction processes of the MT1 and MT2 membrane receptors, pro-oxidant and antioxidant activities, reduction in growth factor uptake, direct inhibition of enzymes that metabolize inactive to active indirect carcinogens, reversal of the Warburg effect, telomerase inhibiting actions, induction of epigenetic processes, anti-inflammation, etc. If there is one, the common denominator for these differential actions has not been revealed. Multiple actions of melatonin are equally as common in normal cells as in cancerous tissues. Because of this, we recently proposed that the basic function of melatonin at the molecular level remains unidentified and what are being measured are merely epiphenomena of melatonin’s fundamental function [73,81]. This speculated unknown site/mechanism of action of melatonin is reminiscent of the Higgs boson of the physical sciences before it was identified.

One of the most extraordinary recent revelations regarding melatonin’s activity in reference to cancer cell biology is its ability to convert cancers that are resistant to radio- or chemotherapy to a therapy-sensitive state. Again, the underpinnings underlying this transformation have escaped identification. It seems one potential explanation for this phenomenon is the capacity of melatonin to synchronize or desynchronize clock processes in cancer cells. The state of molecular metabolic rhythms in individual cells likely changes their response to external stimuli, including drugs. For the same reason, melatonin’s ability to elevate the sensitivity of cancer cells to inhibition by oncostatic drugs may stem from its effects on cellular biological rhythms. Even before the mechanisms involved are clarified, however, it would seem expedient to use melatonin to modulate the sensitivity of cancers to conventional drugs as well as to reduce their toxicity.

Mutated cells that are destined to develop into cancerous tissue are able to flourish in a highly inflamed microenvironment. Additionally, they can avoid immune recognition and limit their immune reactivity [361]. If these newly-defined hallmarks of tumors can be overcome with appropriate countermeasures, the likelihood of a tumor gaining dominance and undergoing cancer progression may be avoided. It is documented that activation of leucocytes in the tumor microenvironment extends the cellular damage already experienced by mutated cells contributing to further neoplastic transformation. The mechanisms that contribute to this augmented damage enhance free radicals and elevate growth and angiogenic factors [362]. Cytokine gene activation by appropriate cells increases the cytokine concentrations in the tumor microenvironment which promotes cellular proliferation and tumor progression [195]. Studies have shown that quelling the local inflammatory response by NF-κB inhibition is an aid to limiting tumor expansion [363,364,365].

That melatonin modulates NF-κB translocation into the nucleus and its binding to DNA has been often observed [366,367,368]. These actions could be relevant to melatonin’s ability to stymie tumor progression since it could influence the redox status and immune microenvironment of the tumor. While melatonin has been reportedly shown to inhibit NF-κB in normal cells, considering the context specificity of the actions of the indoleamine [224], to assume it performs the same function in cancer cells, however, is likely not judicious. Finally, that melatonin suppresses inflammatory responses has been routinely confirmed [368,369,370,371].

Exosomes are small membrane-bound vesicles which are discharged from cells into their microenvironment; these packages contain a wide range of proteins such as mRNAs and miRNAs [372]. Due to their location outside and between cells, they can play an essential role in cell-to-cell communication [373]. By changing the immune status of the microenvironment, exosomes released from tumor cells can impart metastatic information to adjacent normal cells; this information is believed to, in part, aid cancer cells in achieving immune escape [374]. Also in the tumor microenvironment, immune-capable cells including T cells, T regulatory cells (Tregs), dendritic cells, macrophages, and stromal cells are found. These components impact the tumor status as well as the treatment modality of the tumor [375]. Melatonin is known to have significant immunomodulatory potential [369] and can, therefore, influence the cross-talk between immune cells and tumor cells. By this means, melatonin influences tumor cell proliferation, invasion, etc. While the immunological actions of melatonin are well documented under many experimental conditions, its actions on the specific immune cells and exosomes of the tumor microenvironment have gone uninvestigated. In a study designed to examine vesicle release from normal interstitial cells of the seminal vesicle, melatonin influenced shedding of exosomes (also of ectosomes and multivesicular bodies) leading to changes in paracrine signaling of adjacent cells [376]. Considering the significance of the immune system to tumor cell biology, examining the immune state of both cells and exosomes of the microenvironment of tumor cells would be informative in terms of understanding the role of melatonin in determining tumor progression [377].

Cellular suicide due to apoptosis is accepted as a common means by which chemotherapies, ionizing radiation, etc., kill cells. Moreover, once this cell death pathway is initiated and the executioner caspases are activated, it was thought that this process went to completion, i.e., the cell would implode. It seems, however, that some highly resilient cells have only a near death experience because they maintain a lifeline in the event the microenvironment becomes more favorable. Thus, even though some cells may be on the brink of death, they are capable of almost a full recovery if the circumstances in which they exist improve; the newly-discovered process of recovery has been named anastasis.

Anastasis occurs in both cancer cells and normal cells. Cancer cells that survive treatment may give rise to the recurrence of tumors so the treatment, in essence, fails. While the molecular mechanisms governing anastasis are yet to be fully defined [378], there is great interest in identifying molecules that will prevent the recovery of cancer cells allowing them to continue on the apoptosis pathway. Conversely, there is equally strong interest to identify molecules that would hasten anastasis in normal cells allowing them to survive and regain function. For example, revitalizing normal cells that were near death would have important implications for cardiomyocytes that have experienced ischemia/reperfusion injury or neurons and glia in stroke patients.

**Figure 10:**
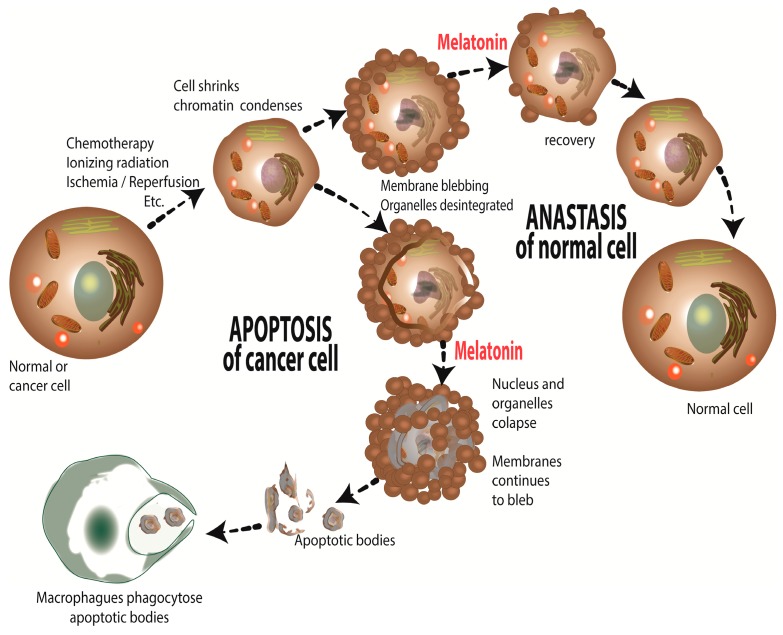
Anastasis, a recently-discovered cellular function, describes the recovery a cell undergoes after apoptosis has been initiated but then the stimulus that launched the apoptosis process is withdrawn. As illustrated here, based on what is known about the context specificity of melatonin’s actions, the function of melatonin on anastasis will differ between normal and cancer cells. Thus, the addition of melatonin to cancer cells at a time that the apoptosis initiates is withdrawn will push cancer cells along the apoptosis pathway, while under the same treatment, normal cells will be induced to recover more quickly.

On the basis of what is known about the context specificity of the actions of melatonin, it seems possible that melatonin may be useful in killing already damaged cancer cells while promoting the survival of injured normal tissue, i.e., both preventing or promoting anastasis depending on the cell type or circumstances (Figure 10). Certainly, the actions of melatonin are known to vary between cancer and normal cells [81,224]. If melatonin is found to have such differential actions in cells that survive the harshest of conditions, it would have important implications in cancer therapies as well as in heart attack and stroke patients, etc.

The collateral toxicity and collateral lethality of chemo- and radiotherapies are serious concerns regarding their use. The damage to normal tissues inflicted by chemotherapies are both acute and chronic. Melatonin, in numerous experimental paradigms, has been shown to mitigate acute, normal cell damage, e.g., oral mucositis due to ionizing radiation [379] as well as the cardio-hepatic and renal toxicity of many drugs [105,380]. Relative to chronic consequences, the cardiac damage resulting from chemotherapy administration may lead to compromised long-term heart function. Since melatonin protects the heart from multiple adverse processes [381,382,383,384,385,386], it is highly likely that chronic, life threatening heart failure may be averted. Despite findings such as these, the application of this information to clinical situations has been questionably and possibly unethically slow. Melatonin is essentially a non-toxic endogenously-produced molecule that is available at a pharmaceutical grade purity for use in humans and it should be put to immediate use, or minimally, immediate testing, in situations where collateral normal tissue damage occurs. There are many individuals who could potentially benefit from such co-treatments.

## Figures and Tables

**Table 1:** Studies in which melatonin administration overcame the resistance of tumors to conventional therapies.

Cancer Type	Cells Resistant to	Proposed Melatonin Action	Reference
Human glioma (A172 and U87MG cells)	Death receptor ligand (TRAIL)	Increased death receptor (DR5) sensitivity	[315]
Human Ewing sarcoma (SK-N-Mc cells)	Vincristine or ifosofamide	Potentiation of extrinsic apoptotic pathway	[316]
Hepatocellular carcinoma (tissue samples from cancer patients)	Various chemotherapies or radiotherapies	Inhibition of survivin and XIAP	[249]
Human lung adenocarcinoma (SK-LU-1 cells)	Cisplatin	Cell cycle arrest in S phase	[260]
Human breast cancer xenografts (MCF-7 cells)	Doxorubicin	Circadian regulated kinase inhibition	[317]
Human breast cancer xenografts (MCF-7 cells)	Tamoxifen	Circadian regulated kinase inhibition	[308]
Human breast cancer (MCF-7 cells)	Ionizing radiation	Downregulation of DNA repair	[222]
Human breast cancer (MCF-7 cells)	Ionizing radiation	Modulation of p53	[223]
